# Extracellular Vesicles Are Associated With Outcome in Veno-Arterial Extracorporeal Membrane Oxygenation and Myocardial Infarction

**DOI:** 10.3389/fcvm.2021.747453

**Published:** 2021-11-04

**Authors:** Patrick M. Siegel, Ileana Bender, Julia Chalupsky, Lukas A. Heger, Marina Rieder, Georg Trummer, Tobias Wengenmayer, Daniel Duerschmied, Christoph Bode, Philipp Diehl

**Affiliations:** ^1^Department of Cardiology and Angiology I, University Heart Center Freiburg – Bad Krozingen, Faculty of Medicine, University of Freiburg, Freiburg, Germany; ^2^Department of Cardiovascular Surgery, University Heart Center Freiburg – Bad Krozingen, Faculty of Medicine, University of Freiburg, Freiburg, Germany

**Keywords:** ECMO - extracorporeal membrane oxygenation, ECLS (VA), survival, myocardial infarction, STEMI, cardiomyocyte, caveolin-3, extracellular vesicle (EV)

## Abstract

**Background:** Veno-arterial extracorporeal membrane oxygenation (VA-ECMO) is being increasingly applied in patients with circulatory failure, but mortality remains high. An inflammatory response syndrome initiated by activation of blood components in the extracorporeal circuit may be an important contributing factor. Patients with ST-elevation myocardial infarction (STEMI) may also experience a systemic inflammatory response syndrome and are at risk of developing cardiogenic shock and cardiac arrest, both indications for VA-ECMO. Extracellular vesicles (EV) are released by activated cells as mediators of intercellular communication and may serve as prognostic biomarkers. Cardiomyocyte EV, released upon myocardial ischemia, hold strong potential for this purpose. The aim of this study was to assess the EV-profile in VA-ECMO and STEMI patients and the association with outcome.

**Methods:** In this prospective observational study, blood was sampled on day 1 after VA-ECMO initiation or myocardial reperfusion (STEMI patients). EV were isolated by differential centrifugation. Leukocyte, platelet, endothelial, erythrocyte and cardiomyocyte (caveolin-3^+^) Annexin V^+^ EV were identified by flow cytometry. EV were assessed in survivors vs. non-survivors of VA-ECMO and in STEMI patients with normal-lightly vs. moderately-severely reduced left ventricular function. Logistic regression was conducted to determine the predictive accuracy of EV. Pearson correlation analysis of EV with clinical parameters was performed.

**Results:** Eighteen VA-ECMO and 19 STEMI patients were recruited. Total Annexin V^+^, cardiomyocyte and erythrocyte EV concentrations were lower (*p* ≤ 0.005) while the percentage of platelet EV was increased in VA-ECMO compared to STEMI patients (*p* = 0.002). Total Annexin V^+^ EV were increased in non-survivors of VA-ECMO (*p* = 0.01), and higher levels were predictive of mortality (AUC = 0.79, *p* = 0.05). Cardiomyocyte EV were increased in STEMI patients with moderately-severely reduced left ventricular function (*p* = 0.03), correlated with CK-MB_max_ (*r* = 0.57, *p* = 0.02) and time from reperfusion to blood sampling (*r* = 0.58, *p* = 0.01). Leukocyte EV correlated with the number of coronary stents placed (*r* = 0.60, *p* = 0.02).

**Conclusions:** Elevated total Annexin V^+^ EV on day 1 of VA-ECMO are predictive of mortality. Increased cardiomyocyte EV on day 1 after STEMI correlate with infarct size and are associated with poor outcome. These EV may aid in the early identification of patients at risk of poor outcome, helping to guide clinical management.

## Introduction

Veno-arterial extracorporeal membrane oxygenation (VA-ECMO) is applied in patients with circulatory failure and its use has increased in recent years (1). Indications include refractory cardiogenic shock, massive pulmonary embolism, cardiac arrest, and failure to wean from cardiopulmonary bypass after cardiac surgery (2–4). The overall survival rate for patients with VA-ECMO is approximately 58% but varies depending on the underlying condition (5). An important factor contributing to high mortality in VA-ECMO patients are ECMO-associated complications such as bleeding (6), thrombosis (7) and a systemic inflammatory response syndrome (8). These complications may in turn be caused by *activation* and alteration of blood components (platelets, leukocytes, complement factors) after contact with the extracorporeal circuit (9). *Extracellular vesicles (EV)* may also be playing an important role, as we recently reported increased levels of EV in VA-ECMO patients compared to healthy controls (10).

EV can be released by most cell types in response to *activation* and consist of a lipid bilayer membrane (11, 12). The term “Extracellular Vesicles” recommended by the International Society for Extracellular Vesicles (ISEV) and the European Society of Cardiology (ESC) for particles released from a cell that are delimited by a lipid bilayer and cannot replicate and is favored over variably defined terms such as “microparticles” and “microvesicles” (13, 14). Depending on their cellular origin, EV contain bioactive molecules, surface receptors and genetic information, e.g., DNA, RNA, and microRNA, which are delivered to their target cells rendering them biologically active *mediators of intercellular communication*. EV may be identified by the presence of phosphatidylserine on their surface which can be detected by Annexin V (15, 16). Moreover, EV express surface-specific antigens from their cells of origin allowing identification by flow cytometry (13). EV are emerging as novel prognostic and diagnostic *biomarkers* in various cardiovascular diseases (17).

Patients with ST-elevation myocardial infarction (STEMI) share several characteristics with VA-ECMO patients, as they often experience a systemic inflammatory response syndrome (18) and are prone to develop cardiogenic shock or cardiac arrest requiring VA-ECMO therapy (19–21). Increased levels of circulating EV have been reported in STEMI patients (22, 23) and EV have also been associated with outcome in these patients (23). An important role could be played by *cardiomyocyte* EV in patients with myocardial infarction. Several *in vitro* studies have shown that cardiomyocyte EV are released from ischemic cardiomyocytes in cell culture and animal models of myocardial infarction (24–26). However, the distribution and concentration of these EV in patients with myocardial infarction requires further investigation.

The aim of this study was to assess the EV-profile in VA-ECMO and STEMI patients and to identify EV associated with outcome as potential novel prognostic biomarkers.

## Materials and Methods

### Recruitment and Management of STEMI Patients

Patients with STEMI were recruited prospectively from the intensive and intermediate care wards of the University Hospital in Freiburg, Germany, from April 2020 until March 2021. Patients were identified by daily screening of the electronic patient data management system. This study adhered to the definition of STEMI by the European Society of Cardiology and patients were treated as per guideline recommendations (27). All STEMI patients received unfractionated heparin. Patients with STEMI who had either full vessel occlusion or high-grade stenosis in the coronary angiogram requiring coronary intervention were eligible. Exclusion criteria were: (A) Age <18 or >80 years, (B) hematological malignancies, (C) Hemoglobin (Hb)-values under 8 g/dl, Sepsis (as defined by positive blood cultures, D), cardiac arrest (E), mechanical circulatory support (e.g., VA-ECMO, Impella) (F). After discarding the first tube, citrated blood was drawn slowly after antecubital vein puncture at one time point 2–22 h after reperfusion and carefully transferred to the laboratory for EV isolation and analysis.

### Recruitment and Management of VA-ECMO Patients

Patients receiving VA-ECMO were recruited prospectively from the intensive care units of the medical and heart surgical intensive care wards of the University Hospital in Freiburg, Germany from December 2019 until December 2020. Daily screening of the patient data management system identified patients on VA-ECMO. Patients were eligible if they were receiving VA-ECMO. Exclusion criteria were: (A) Age <18 or >80 years, (B) hematological malignancies and (C) a hemoglobin value <8 g/dl. After discarding the first tube, citrated blood was carefully drawn from an arterial line from patients 2–24 h after VA-ECMO initiation (=day 1). Blood was carefully transported to the laboratory where EV isolation and analysis were performed. Clinical and laboratory parameters were obtained from the electronic patient data management system.

The decision on the placement of VA-ECMO was made by an experienced ECMO physician while implantation and management were carried out as described previously (28, 29). For most patients, the diameter of venous cannulas was 21–23 F and 15–17 F for arterial cannulas. Patients received unfractionated heparin with a partial thromboplastin time (PTT) target of 40–50 s. If patients had signs of bleeding or thrombosis, individual PTT-targets were set by an experienced ECMO physician. VA-ECMO was carried out using the Stöckert® centrifugal pump (LivaNova PLC, London, United Kingdom), the Maquet Cardiohelp System with an HLS Set Advanced (Maquet Cardiopulmonary GmbH, Rastatt, Germany), the CARL system (Resuscitec, Freiburg, Germany) or the Deltastream system (Xenios AG, Heilbronn, Germany).

### Isolation and Storage of Extracellular Vesicles

20 ml of citrated blood were transferred to the laboratory at room temperature taking care to avoid agitation and EV isolation was started within 1.5 h after blood sampling. EV were isolated and stored as described previously (30). Samples with macroscopic signs of hemolysis were discarded. Samples that were used for flow cytometric analysis had only been frozen and defrosted once. After defrosting, 2–3 tubes with EV (each containing 100 μl EV diluted in RPMI buffer) were pooled and vortexed before being prepared for flow cytometry.

### Flow Cytometry

The following antibodies were used: PE Annexin V (Biolegend, USA), APC anti-CD61 (clone VI-PL2, Biolegend, USA), Brilliant Violet (BV) 421 anti-CD62E (clone 68-5H11, BD Biosciences, USA), APC anti-CD45 (clone HI-30, Biolegend, USA), PE/Cy7 anti-CD235a (clone HI264, Biolegend, USA), Alexa Fluor (AF) 647 anti-caveolin-3 (clone A3, Santa Cruz Biotechnology Inc., USA). Matching isotype control antibodies were ordered from the same company as the binding antibodies.

FACS tubes were prepared with PE Annexin V and a single specific antibody or a corresponding isotype control. This single stain strategy was adopted as it reduced the overall brightness of the sample, reducing the need for compensation and the risk of “false-positive” events and allowing a more specific EV analysis. 2.5 μl of PE Annexin V were added to every tube. The following volumes of antibodies (or their isotype controls) were added to the respective single stain tubes: APC anti-CD61: 1 μl; BV421 anti-CD62E: 5 μl; APC anti-CD45: 1 μl; PE/Cy7 anti CD235a: 5 μl; AF 647 anti-caveolin-3: 5 μl. 20 μl of EV were added to each tube and staining was allowed (20 min, RT, dark). Afterwards, 400 μl of Annexin V binding buffer was added to each tube followed by vortexing and samples were incubated for 10 min in the dark. Samples were recorded at low flow rate using a FACS Canto II Flow Cytometer (BD, USA). The lower detection limit was placed as a threshold above the electronic background noise of the flow cytometer and an appropriate SSC threshold was set. 10,000 Trucount^TM^ (BD, USA) beads were recorded per sample. Absolute EV counts are presented per ml of diluent in the FACS tube and were determined using BD Trucount^TM^ Tubes following the manufacturer's instructions. Total Annexin V^+^ EV/ml were quantified in the first sample for every patient stained only with PE Annexin V^+^.

A gate including EV between 100 nm and 1 μm (= “microvesicles”) was set and verified for each patient using size reference beads (Flow Cytometry Sub-micron Particle Size Reference Kit, Thermo Fisher Scientific, USA). Gating was then performed for Annexin V^+^ events. Platelet (Annexin V^+^ CD61^+^), leukocyte (Annexin V^+^ CD45^+^), cardiomyocyte (Annexin V^+^ caveolin-3^+^), endothelial (Annexin V^+^ CD62E^+^) and erythrocyte EV (Annexin V^+^ CD235a^+^) were identified. Flow cytometric data was analyzed using FlowJo V10.6.0 (FlowJo, LLC). Percentages of Annexin V^+^ EV subpopulations were calculated by dividing the absolute concentration of the EV subpopulation by the concentration of the total Annexin V^+^ EV population and multiplying by 100.

### Laboratory Parameters

Laboratory parameters were taken from the electronic patient data management system closest to the time of blood sampling. The IL-6 concentration was analyzed in the supernatant remaining after the second centrifugation step in the EV isolation protocol. An Elecsys IL-6 test run on a Roche cobas 8000 – e801 module (Roche, Switzerland) was used to determine the IL-6 concentration.

### Statistics

Variables are presented as mean±SEM or median (interquartile range). Unpaired *t*-tests were conducted to analyze differences of means. Simple logistic regression analysis was carried out to determine association of EV with mortality in VA-ECMO patients. Areas under the receiver operating characteristics (ROC) curve (AUC) were calculated to determine predictive accuracy of these parameters. The Pearson correlation coefficient was calculated to determine the correlation between two parameters. A *p*-value ≤ 0.05 was considered statistically significant. Statistical analysis was performed using GraphPad Prism V9.1 (GraphPad Software, San Diego, California, USA).

## Results

### Clinical Characteristics

Eighteen patients receiving VA-ECMO and 19 patients with STEMI were recruited for this study (Table 1). The median age of VA-ECMO patients was 63 years, the median age of STEMI patients was 60 years. There were 4 females in the VA-ECMO group and 7 females in the STEMI group. Patients received VA-ECMO for cardiogenic shock (15 patients) or during extracorporeal cardiopulmonary resuscitation (eCPR, 3 patients). The median time on VA-ECMO was 5 days. Twelve VA-ECMO patients survived until discharge from the intensive care wards and were considered “survivors”. Of the 6 non-survivors, 5 patients died of the devastating underlying disease and one patient died of a severe ECMO-induced coagulopathy and bleeding complications. Eighteen STEMI patients survived until discharge from the intensive care wards. Compared to STEMI patients, which did not suffer from bleeding or thrombotic complications, 8 VA-ECMO patients had severe bleeding and 2 had thrombotic complications. Eleven VA-ECMO patients suffered from coronary artery disease (CAD). STEMI patients had more cardiovascular risk factors than VA-ECMO patients, and all STEMI patients received dual anti-platelet therapy (DAPT) compared to 7 VA-ECMO patients. Moreover, all VA-ECMO patients were on mechanical ventilation as opposed to no patients in the STEMI group. As expected, VA-ECMO patients had higher levels of CRP, lactate, creatinine, and urea, but a lower prothrombin time ratio, platelet count and Hb-concentration. The number of diseased vessels, the number of coronary stents placed, the anti-platelet agents applied and the time from reperfusion to blood sampling for STEMI patients can be found in Supplementary Table S1.

**Table 1:** Clinical characteristics of STEMI and VA-ECMO patients.

	**VA-ECMO**	**STEMI**	***p-*value**
Patients, *n* (%)	18	19	
Female, *n* (%)	4 (22)	7 (37)	0.48
Survivors, *n* (%)	12 (67)	18 (95)	**0.04**
Age, y (Q1-Q3)	63.0 (52.8–71.5)	60.0 (53.0–69.0)	0.75
Coronary Artery Disease, *n* (%)	11 (61)	19 (100)	**0.003**
Atrial fibrillation, *n* (%)	2 (11)	1 (5)	0.60
Diabetes mellitus, *n* (%)	2 (11)	3 (16)	>0.99
Hypertension, *n* (%)	1 (6)	12 (63)	** <0.001**
Active smoker, *n* (%)	2 (11)	12 (63)	**0.002**
Hypercholesterolemia, *n* (%)	4 (22)	9 (47)	0.17
Cancer, *n* (%)	0 (0)	1 (5)	>0.99
Acute renal failure, *n* (%)	4 (22)	1 (5)	0.18
Acute liver failure, *n* (%)	1 (6)	0 (0)	0.49
Catecholamines, *n* (%)	16 (89)	1 (5)	** <0.001**
Dual anti-platelet therapy, *n* (%)	7 (39)	19 (100)	**<0.001**
Major bleeding, *n* (%)	8 (44)	0 (0)	**0.001**
Thrombotic events, *n* (%)	2 (11)	0 (0)	0.23
Mechanical ventilation, *n* (%)	18 (100)	0 (0)	** <0.001**
PEEP (mbar, Q1-Q3)	8.0 (6.5–12.8)		
F_i_O (%, Q1-Q3)	50.0 (40.0–50.0)		
Respiratory rate (/min, Q1-Q3)	14.5 (11.8–16.0)		
p_a_O_2_ (mmHg, Q1-Q3)	116.5 (86.8–180.3)		
p_a_CO_2_ (mmHg, Q1-Q3)	41.1 (37.3–43.4)		
**Type of VA-ECMO**, ***n*** **(%)** Stöckert Sorin Maquet Deltastream CARL	7 (39) 8 (44) 2 (11) 1 (6)		
**Indications for VA-ECMO**, ***n*** **(%)**			
Cardiogenic shock - Postoperative - Myocardial Infarction - Cardiomyopathy - Endocarditis - Pulmonary embolism	15 (83) 7 (39) 2 (11) 4 (22) 1 (6) 1 (6)		
eCPR	3 (17)		
Days on ECMO (d, Q1-Q3)	5.0 (3.0–7.0)		
ECMO Blood Flow (l/min, Q1-Q3)	4.5 (3.3–4.9)		
WBC (x10^3^ /μl, Q1-Q3)	9.0 (5.9–11.6)	11.3 (9.3–13.0)	0.51
Hb (g/dl, Q1-Q3)	8.6 (8.0–10.0)	14.2 (13.4–15.2)	** <0.001**
Platelets (x10^3^ /μl, Q1-Q3)	105 (60.0–132.3)	217.0 (192.0–246.0)	** <0.001**
Creatinine (mg/dl, Q1-Q3)	1.5 (1.0–2.4)	1.0 (0.9–1.1)	**0.001**
Urea (mg/dl, Q1-Q3)	54 (34.0–85.0)	29.0 (26.0–33.0)	** <0.001**
Bilirubin (mg/dl, Q1-Q3)	1.9 (1.2–3.3)	0.6 (0.4–0.9)	0.07
AST (U/l, Q1-Q3)	182.0 (109.5–477.0)	181.5 (93.0–468.0)	0.48
ALT (U/l, Q1-Q3)	56.5 (35.3–99.3)	45.5 (28.3–81.5)	0.26
CRP (mg/l, Q1-Q3)	35.7 (20.4–94.2)	3.1 (1.0–12.0)	**0.02**
IL-6 (pg/ml, Q1-Q3)	43.3 (15.6–405.3)	42.2 (19.0–162.8)	0.33
Lactate (mmol/l, Q1-Q3)	2.9 (1.3–4.7)	1.8 (1.1-2.2)	**0.02**
Prothrombin time ratio (%, Q1-Q3)	61.0 (46.8–73.0)	90.5 (81.5–106.5)	** <0.001**
PTT (s, Q1-Q3)	47.5 (41.5–66.8)	38.0 (27.5–85.0)	0.89
CK_max_ (U/L, Q1-Q3)		2,585 (1,296–4,114)	
CK-MB_max_ (U/L, Q1-Q3)		363.0 (182.5–446.3)	
Total Cholesterol (mg/dl, Q1-Q3)		189.0 (144.0–222.0)	
LDL cholesterol (mg/dl, Q1-Q3)		128.0 (94.0–149.0)	
HDL cholesterol (mg/dl, Q1-Q3)		49.0 (40.0–54.0)	
HbA1c (%, Q1-Q3)		5.6 (5.4–6.1)	

*Data are presented as median (interquartile range, Q1-Q3) or number of patients (%). Denominator of the percentage is the total number of subjects in the group. p-values were calculated by an unpaired t-test or a Fisher's exact test. Significant p-values are indicated in bold. “Cardiomyopathy” includes ischemic cardiomyopathies and cardiomyopathies of unknown origin. Several parameters were only available for either VA-ECMO or STEMI patients. Laboratory values were taken from the data management system closest to the time point of blood sampling. Criteria published by the International Society on Thrombosis and Haemostasis were used to define major bleeding (31). ALT, alanine aminotransferase; AST, aspartate aminotransferase; CK_max_, maximum value of creatine kinase; CK-MB_max_, maximum value of creatine kinase MB. CRP, C-reactive protein; eCPR, extracorporeal cardiopulmonary resuscitation; F_i_O, fraction of inspired oxygen; Hb, hemoglobin; IL-6, Interleukin 6; HDL, High density lipoprotein; LDL, Low density lipoprotein; PEEP, positive end-expiratory pressure; PTT, partial thromboplastin time; WBC, white blood cells*.

### The EV-Profile Differs Significantly Between STEMI and VA-ECMO Patients

**Figure 1:**
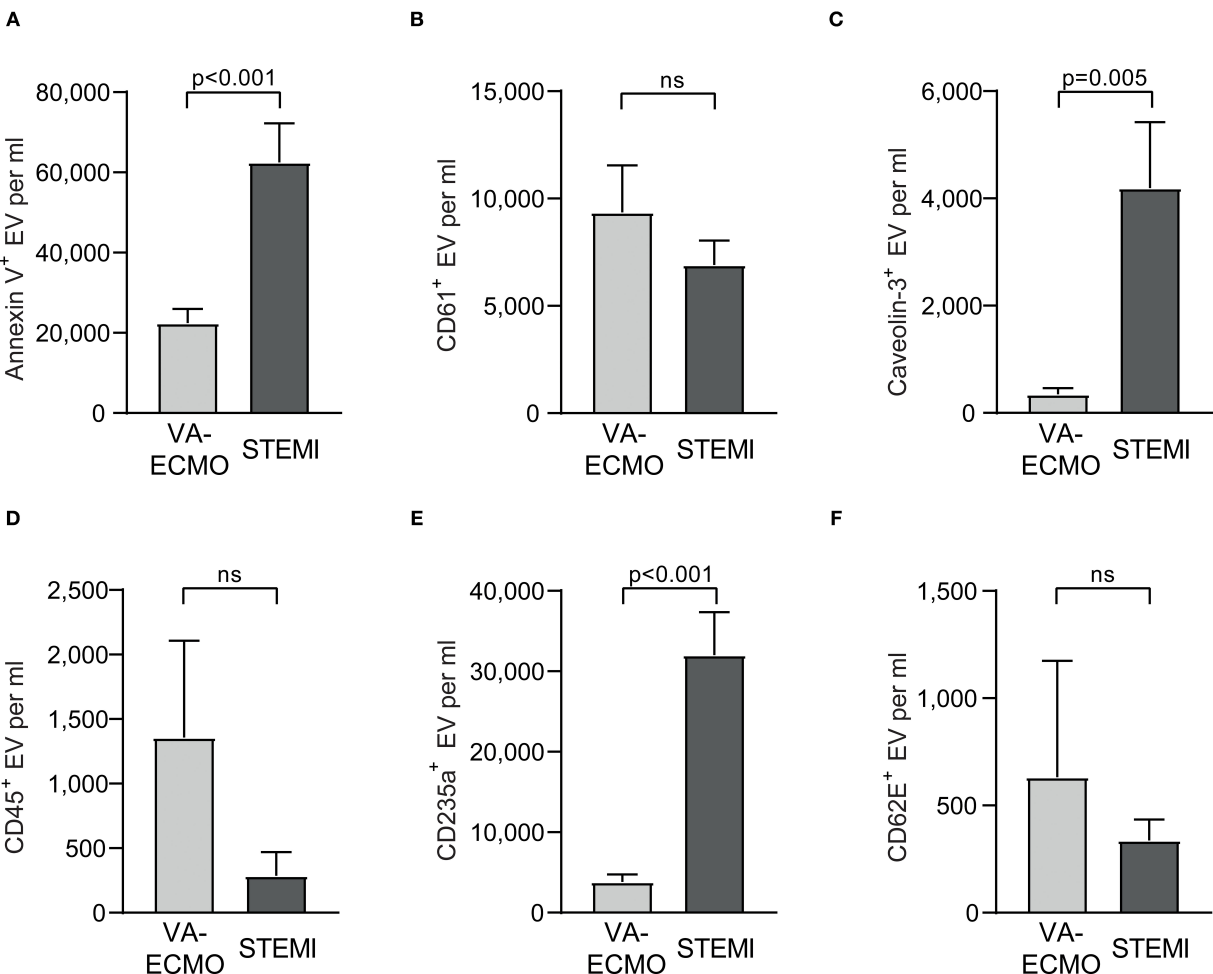
Extracellular vesicle (EV) concentration in patients receiving veno-arterial extracorporeal membrane oxygenation (VA-ECMO) compared to patients with ST-elevation myocardial infarction (STEMI). **(A)** Total Annexin V^+^ EV/ml, **(B)** CD61^+^ (platelet) EV/ml, **(C)** caveolin-3^+^ (cardiomyocyte) EV/ml, **(D)** CD45^+^ (leukocyte) EV/ml, **(E)** CD235a^+^ (erythrocyte) EV/ml, **(F)** CD62E^+^ (endothelial) EV/ml. EV isolation, analysis and quantification were performed as described in the Materials and Methods section. Data are presented as mean±SEM. p-values were calculated by an unpaired Student's t-test, *p* ≤ 0.05 was considered significant, ns – not significant.

When assessing the absolute EV concentrations in VA-ECMO and STEMI patients, several differences were observed. Total Annexin V^+^ EV/ml were significantly higher in STEMI patients compared to VA-ECMO patients (VA-ECMO vs. STEMI: 22,449 ± 3,519 vs. 62,482 ± 9,781, *p* < 0.001, Figure 1). Caveolin-3^+^ EV/ml were also significantly higher in STEMI patients, as were CD235a^+^ EV/ml (VA-ECMO vs. STEMI, caveolin-3^+^ EV/ml: 347.0 ± 113.4 vs. 4,191 ± 1,231, *p* = 0.005; CD235a^+^ EV/ml: 3,807 ± 933.1 vs. 32,033 ± 5,323, *p* < 0.001). The concentration of CD61^+^ EV/ml, CD45^+^ EV/ml and CD62E^+^ EV/ml were not significantly different between STEMI and VA-ECMO patients (VA-ECMO vs. STEMI, CD61^+^ EV/ml: 9,356 ± 2,182 vs. 6,907 ± 1,143, *p* = 0.32; CD45^+^ EV/ml: 1,357 ± 751.2 vs. 286.4 ± 184.1, *p* = 0.19; CD62E^+^ EV/ml: 632.2 ± 542.3 vs. 336.4 ± 98.0, *p* = 0.57).

**Figure 2:**
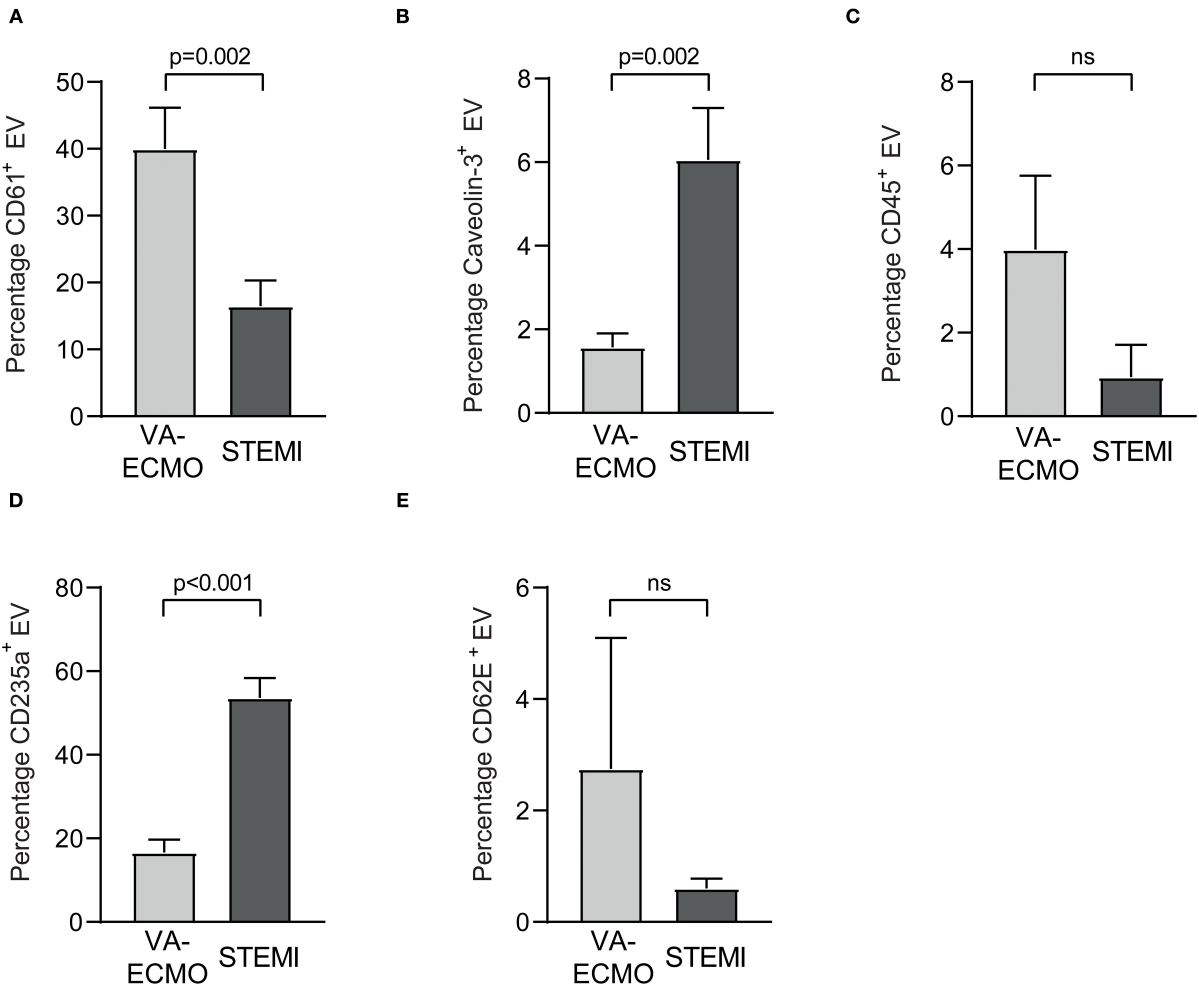
Extracellular vesicles (EV) as percentage of Annexin V^+^ EV from patients receiving veno-arterial extracorporeal membrane oxygenation (VA-ECMO) compared to patients with ST-elevation myocardial infarction (STEMI). **(A)** CD61^+^ (platelet) EV, **(B)** caveolin-3^+^ (cardiomyocyte) EV, **(C)** CD45^+^ (leukocyte) EV, **(D)** CD235a^+^ (erythrocyte) EV, **(E)** CD62E^+^ (endothelial) EV. EV isolation, analysis and quantification were performed as described in the Materials and Methods section. Data are presented as mean±SEM. *p*-values were calculated by an unpaired Student's *t*-test, *p* ≤ 0.05 was considered significant, ns, not significant.

The percentages of the Annexin V^+^ EV subpopulations also differed significantly. The percentage of CD61^+^ EV was significantly higher in VA-ECMO patients compared to STEMI patients (VA-ECMO vs. STEMI, percentage of CD61^+^ EV: 40.0 ± 6.2 vs. 16.5 ± 3.8, *p* = 0.002, Figure 2). The percentages of caveolin-3^+^ EV and CD235a^+^ EV were significantly lower in VA-ECMO patients compared to STEMI patients (VA-ECMO vs. STEMI, percentage of caveolin-3^+^ EV: 1.5 ± 0.3 vs. 6.1 ± 1.2, *p* = 0.002; percentage of CD235a^+^ EV: 16.6 ± 3.1 vs. 53.6 ± 4.8, *p* < 0.001). The percentages of CD45^+^ EV and CD62E^+^ EV did not differ significantly between VA-ECMO and STEMI patients (VA-ECMO vs. STEMI, percentage of CD45^+^ EV: 4.0 ± 1.8 vs. 0.9 ± 0.7, *p* = 0.13; percentage of CD62E^+^ EV: 2.8 ± 2.4 vs. 0.6 ± 0.2, *p* = 0.36). Representative populations of total Annexin V^+^, CD61^+^, caveolin-3^+^, CD235a^+^, CD45^+^ and CD62E^+^ EV identified by flow cytometry are presented in Supplementary Figure S1.

### The EV Profile Is Similar in VA-ECMO Patients With and Without CAD

To account for different underlying diseases in VA-ECMO patients, subgroup analysis was carried out. EV levels were compared between VA-ECMO patients with CAD (*n* = 11) and those without CAD (*n* = 7). No significant differences in absolute EV levels or percentages were found between the two groups (Supplementary Figures S2, S3).

Moreover, EV levels in the subgroup of VA-ECMO patients with CAD were compared with EV levels in STEMI patients. Significant differences regarding absolute EV levels and percentages remained as previously observed for the whole VA-ECMO group (Supplementary Figures S4, S5).

### Increased Levels of Total Annexin V^+^ EV/ml on Day 1 of VA-ECMO Therapy Are Predictive of Mortality

Levels of total Annexin V^+^ EV/ml, CD61^+^ EV/ml and CD45^+^ EV/ml blood were significantly increased in non-survivors on day 1 of VA-ECMO therapy (Table 2). Logistic regression analysis revealed that increased levels of total Annexin V^+^ EV/ml on day 1 of VA-ECMO were predictive of mortality (AUC: 0.79, *p* = 0.05). However, CD61^+^ EV/ml and CD45^+^ EV/ml did not achieve significance (AUC: CD61^+^ EV/ml: 0.72, *p* = 0.13, CD45^+^ EV/ml: 0.55, *p* = 0.76). Levels of caveolin-3^+^ EV/ml, CD62E^+^ EV/ml and CD235^+^ EV/ml did not differ significantly between survivors and non-survivors of VA-ECMO therapy. This was also the case for the percentages of Annexin V^+^ EV subpopulations (Table 2).

**Table 2:** Extracellular vesicle (EV) concentration and percentage [%] of Annexin V^+^ EV in survivors and non-survivors of VA-ECMO therapy.

**Parameter**	**Survivor**	**Non-survivor**	***p*-value**
Total Annexin V^+^ EV/ml	16,451 ± 2,506	34,447 ± 7,449	**0.01**
CD61^+^ EV/ml	6,079 ± 1,081	15,912 ± 5,524	**0.03**
Caveolin-3^+^ EV/ml	230.9 ± 67.43	559.7 ± 293.1	0.87
CD45^+^ EV/ml	345.4 ± 217.8	3,380 ± 2,044	**0.05**
CD62E^+^ EV/ml	871.8 ± 789.3	105.0 ± 39.2	0.53
CD235a^+^ EV/ml	2,750 ± 822.9	5,922 ± 2,135	0.11
CD61^+^ EV [%]	38.4 ± 5.0	43.1 ± 16.7	0.73
Caveolin-3^+^ EV [%]	1.7 ± 0.4	1.4 ± 0.5	0.70
CD45^+^ EV [%]	2.1 ± 0.8	7.9 ± 4.9	0.12
CD62E^+^ EV [%]	4.1 ± 3.6	0.2 ± 0.1	0.45
CD235a^+^ EV [%]	18.0 ± 4.2	13.9 ± 3.9	0.54

*CD61^+^ (platelet) EV, caveolin-3^+^ (cardiomyocyte) EV, CD45^+^ (leukocyte) EV, CD62E^+^ (endothelial) EV, CD235a^+^ (erythrocyte) EV. Data are presented as mean±SEM. p-values were calculated by an unpaired Student's t-test. Significant p-values are indicated in bold*.

### Caveolin 3^+^ EV Are Elevated in STEMI Patients Compared to Patients With Stable CAD

To further characterize the distribution of cardiomyocyte EV in other patient groups, caveolin-3^+^ EV were analyzed in 10 patients with stable coronary artery disease and compared to STEMI patients (Supplementary Figure S6). Clinical characteristics of patients with stable CAD are described in the caption to Supplementary Figure S6. The level of caveolin 3^+^ EV per ml and the percentage of caveolin 3^+^ EV were significantly elevated in STEMI patients compared to patients with stable CAD, whereas the level of Annexin V^+^ EV did not differ significantly.

### Elevated Levels of Caveolin-3^+^ EV/ml Are Associated With Moderately to Severely Reduced Left Ventricular Ejection Fraction After STEMI

To determine whether EV were associated with outcome in STEMI patients, levels of EV/ml and percentages of Annexin V^+^ EV were compared between patients with a left ventricular ejection fraction (LVEF) ≥40% (normal-lightly reduced) and those with an LVEF <40% (moderately-severely reduced) in the first echocardiography after STEMI (Table 3). Caveolin-3^+^ EV/ml were significantly increased in patients with moderately to severely reduced LVEF (*p* = 0.03). All other EV parameters investigated did not show a significant difference between groups.

**Table 3:** Extracellular vesicle (EV) concentration and percentage [%] of Annexin V^+^ EV in patients with ST-elevation myocardial infarction (STEMI) with either normal-lightly (≥40 %) or moderately-severely (<40 %) reduced left ventricular ejection fraction (LVEF).

**Parameter**	**LVEF ≥ 40 % (*n* = 15)**	**LVEF <40 % (*n* = 4)**	***p*-value**
Total Annexin V^+^ EV/ml	57,145 ± 10,360	82,498 ± 26,057	0.30
CD61^+^ EV/ml	7,499 ± 1,408	4,687 ± 645.6	0.33
Caveolin-3^+^ EV/ml	2,819 ± 776.6	8,992 ± 4,425	**0.03**
CD45^+^ EV/ml	300.0 ± 230.9	236.7 ± 219.6	0.89
CD62E^+^ EV/ml	304.2 ± 121.7	449.1 ± 123.0	0.56
CD235a^+^ EV/ml	29,281 ± 5,687	42,353 ± 14,021	0.33
CD61^+^ EV [%]	19.0 ± 4.7	7.0 ± 1.8	0.21
Caveolin-3^+^ EV [%]	5.0 ± 0.9	9.8 ± 4.7	0.11
CD45^+^ EV [%]	1.1 ± 1.0	0.3 ± 0.2	0.68
CD62E^+^ EV [%]	0.6 ± 0.2	0.7 ± 0.2	0.84
CD235a^+^ EV [%]	53.8 ± 5.3	52.8 ± 12.2	0.93

*CD61^+^ (platelet) EV, caveolin-3^+^ (cardiomyocyte) EV, CD45^+^ (leukocyte) EV, CD62E^+^ (endothelial) EV, CD235a^+^ (erythrocyte) EV. LVEF was obtained from the first echocardiography performed by a specialist after STEMI, usually 2-4 days after the event. Data are presented as mean±SEM. p-values were calculated by an unpaired Student's t-test. Significant p-values are indicated in bold*.

### Caveolin-3^+^ EV Correlate With CK-MB_max_ and Time From Reperfusion to Blood Sampling

To further explore the potential of caveolin-3^+^ EV as a biomarker for the severity of myocardial infarction, the correlation of EV with CK-MB_max_, an established marker related to infarct size and outcome after myocardial infarction (32), was determined (Table 4). There was a significant correlation of caveolin-3^+^ EV/ml (*r* = 0.57, *p* = 0.02) and the percentage of caveolin-3^+^ EV (*r* = 0.50, *p* = 0.04) with CK-MB_max_.

**Table 4:** Correlation of extracellular vesicle (EV) concentration and percentage [%] of Annexin V^+^ EV with CK-MB_max_ in STEMI patients.

**Parameter**	***r***	***p*-value**
Total Annexin V^+^ EV/ml	0.38	0.12
CD61^+^ EV/ml	−0.09	0.72
Caveolin-3^+^ EV/ml	0.57	**0.02**
CD45^+^ EV/ml	−−0.17	0.57
CD62E^+^ EV/ml	0.04	0.87
CD235a^+^ EV/ml	0.20	0.43
CD61^+^ EV [%]	−−0.26	0.30
Caveolin-3^+^ EV [%]	0.50	**0.04**
CD45^+^ EV [%]	−−0.25	0.39
CD62E^+^ EV [%]	−−0.08	0.77
CD235a^+^ EV [%]	−−0.16	0.54

*CD61^+^ (platelet) EV, caveolin-3^+^ (cardiomyocyte) EV, CD45^+^ (leukocyte) EV, CD62E^+^ (endothelial) EV, CD235a^+^ (erythrocyte) EV. The Pearson correlation coefficient is presented. Significant p-values are indicated in bold*.

Like other markers of myocardial damage, caveolin-3^+^ EV/ml and the percentage of caveolin-3^+^ EV showed a time dependent increase after reperfusion (Table 5) as they correlated with time from reperfusion to blood sampling (caveolin-3^+^ EV/ml: *r* = 0.58, *p* = 0.01; percentage of caveolin-3^+^ EV: *r* = 0.51, *p* = 0.03). CD235a^+^ MV/ml also showed a correlation with time from reperfusion to blood sampling (*r* = 0.47, *p* = 0.04).

**Table 5:** Correlation of extracellular vesicle (EV) concentration and percentage [%] of Annexin V^+^ EV with time from reperfusion to blood sampling.

**Parameter**	***r***	***p*-value**
Total Annexin V^+^ EV/ml	0.14	0.58
CD61^+^ EV/ml	−−0.01	0.96
Caveolin-3^+^ EV/ml	0.58	**0.01**
CD45^+^ EV/ml	−−0.03	0.91
CD62E^+^ EV/ml	−−0.16	0.53
CD235a^+^ EV/ml	0.47	**0.04**
CD61^+^ EV [%]	−−0.08	0.75
Caveolin-3^+^ EV [%]	0.51	**0.03**
CD45^+^ EV [%]	−−0.09	0.76
CD62E^+^ EV [%]	−−0.25	0.33
CD235a^+^ EV [%]	0.21	0.38

*Blood was sampled from STEMI patients 4–22 h after interventional reperfusion. CD61^+^ (platelet) EV, caveolin-3^+^ (cardiomyocyte) EV, CD45^+^ (leukocyte) EV, CD62E^+^ (endothelial) EV, CD235a^+^ (erythrocyte) EV. The Pearson correlation coefficient is presented. Significant p-values are indicated in bold*.

### CD45^+^ EV Correlate With Number of Coronary Stents Placed

The correlation between the number of coronary stents placed during the acute coronary angiography and EV/ml and the percentages of Annexin V^+^ EV subpopulations was analyzed in STEMI patients (Table 6). CD45^+^ EV/ml and the percentage of CD45^+^ EV showed a significant correlation with the number of coronary stents placed (CD45^+^ EV/ml: *r* = 0.60, *p* = 0.02; percentage of CD45^+^ EV: *r* = 0.65, *p* = 0.01).

**Table 6:** Correlation of extracellular vesicle (EV) concentration and percentage [%] of Annexin V^+^ EV with the number of coronary stents implanted during the acute coronary angiography in STEMI patients.

**Parameter**	***r***	***p*-value**
Total Annexin V^+^ EV/ml	−−0.29	0.23
CD61^+^ EV/ml	0.05	0.83
Caveolin-3^+^ EV/ml	−−0.29	0.24
CD45^+^ EV/ml	0.60	**0.02**
CD62E^+^ EV/ml	−−0.33	0.18
CD235a^+^ EV/ml	−−0.23	0.34
CD61^+^ EV [%]	0.29	0.24
Caveolin-3^+^ EV [%]	−−0.34	0.17
CD45^+^ EV [%]	0.65	**0.01**
CD62E^+^ EV [%]	−−0.25	0.31
CD235a^+^ EV [%]	−−0.23	0.34

*A maximum of 4 stents were placed. CD61^+^ (platelet) EV, caveolin-3^+^ (cardiomyocyte) EV, CD45^+^ (leukocyte) EV, CD62E^+^ (endothelial) EV, CD235a^+^ (erythrocyte) EV. The Pearson correlation coefficient is presented. Significant p-values are indicated in bold*.

## Discussion

In this study, we investigated the EV profile of VA-ECMO and STEMI patients and suggest novel EV biomarker candidates associated with outcome. To the best of our knowledge, this is the first study to compare the EV profiles of VA-ECMO and STEMI patients. Several studies have investigated EV in STEMI patients (22, 23, 33, 34), but data on EV levels and distribution in VA-ECMO patients is limited. Often, results were generated ex-vivo using artificial blood pumps in an experimental setting (35–37). Moreover, previous studies analyzing EV in ECMO patients featured healthy volunteers (10, 38) which share only very few characteristics with VA-ECMO patients. STEMI patients, however, are at risk of developing cardiogenic shock, which may be an indication for VA-ECMO therapy. Moreover, they share several of the underlying risk factors and clinical characteristics with ECMO patients. STEMI patients may therefore be considered ‘more realistic' controls than healthy volunteers.

Interestingly, STEMI patients had elevated absolute levels of total Annexin V^+^ EV compared to VA-ECMO patients. This is in line with previous reports of elevated EV concentrations in STEMI patients compared to other patients with vascular inflammation, e.g., NSTEMI patients, patients recovering from STEMI and patients with stable angina (22, 39–42). Elevated EV levels most likely reflect the release of MV during the thrombotic process, from activated cells and from the ischemic myocardium after reperfusion (17, 43). As opposed to STEMI patients, levels of EV in VA-ECMO patients are influenced by several factors including flow rate, the composition of the extracellular circuit and the underlying disease (10, 36, 44). For example, levels of Annexin V^+^ EV rise with increasing flow rates and the composition of EV is affected by which cells adhere to the different types of extracorporeal circuits and thus, may secrete EV.

Conventional markers associated with outcome in VA-ECMO patients include laboratory parameters such as pH, lactate and bicarbonate and clinical parameters, such as age, the underlying condition and multiorgan failure. Several of these markers have been combined to create prediction models for VA-ECMO patients (45, 46). However, decision making for an individual patient is still unreliable based on these scores alone. Extracellular vesicles hold promise as novel prognostic biomarkers. For example, Annexin V^+^ EV have been suggested as prognostic biomarkers in cardiovascular disease (47, 48), but also cancer (49, 50). In line with this data, we found increased levels of total Annexin V^+^ EV in patients receiving VA-ECMO to be predictive of mortality. To the best of our knowledge, this is the first study to provide a link between increased Annexin V^+^ EV and mortality in these patients. As VA-ECMO is a resource-heavy therapy, early measurement of total Annexin V^+^ EV on day 1 after VA-ECMO initiation, as conducted in our study, may aid clinicians in prognostication and decision making for this patient collective in the future.

We found significantly increased levels of CD235a^+^ erythrocyte EV in STEMI patients, as previously reported (51). The authors suggested that the increased CD235a^+^ EV in STEMI patients were due to intravascular thrombus formation during acute myocardial infarction. CD235a^+^ EV are not only released by growing thrombi, thereby acting as potential *biomarkers* but also contribute actively, as *bioactive mediators*, to thrombus formation by activating the intrinsic and extrinsic pathways (52). CD235a^+^ EV/ml also correlated with the time from reperfusion to blood sampling in STEMI patients. This could indicate an ongoing thrombotic mass even after reperfusion associated with poor outcome (34). Lower levels of CD235a^+^ EV observed in in VA-ECMO patients may therefore explain, at least partly, why thrombotic complications in these patients are less common than bleeding complications (7).

We report similar absolute CD61^+^ platelet EV counts in VA-ECMO and STEMI patients, whereas the percentage of platelet EV was significantly higher in VA-ECMO patients. Increased levels of platelet EV in VA-ECMO patients are likely due to high shear stress occurring in the ECMO circuit which has been previously reported for other forms of extracorporeal circulation (44). Contributing to comparably lower levels of platelet EV in STEMI patients may have been the high rate of dual anti-platelet therapy, known to decrease platelet activation and thereby EV release (16). Additionally, erythrocyte EV, as opposed to platelet EV, seem to be emerging as the predominant EV fraction in STEMI patients (34, 51, 52). Platelet EV are not only a consequence of platelet activation, but are also involved in several biological processes, for example platelet EV can bind to monocytes and induce the release of pro-inflammatory mediators, e.g. IL-1β, TNFα and MCP-1 (53, 54). Platelet EV are therefore likely to be exerting pro-inflammatory effects on other immune cells contributing to the overshooting inflammatory response observed in VA-ECMO patients. Potentially related to their important biological functions, platelet EV have been used as prognostic biomarkers. For example, a recent study found increased levels of platelet EV in patients with STEMI that had progressed to cardiogenic shock compared to those without cardiogenic shock (55). Moreover, platelet EV were also associated with outcome in our study, as we report increased levels of platelet EV in non-survivors of VA-ECMO therapy.

Another important finding of this study was that the level of caveolin-3^+^ cardiomyocyte EV was significantly higher in STEMI patients compared to VA-ECMO patients *and* patients with stable CAD. Caveolin-3 has previously been used as a marker for cardiomyocyte EV (25, 26). Previous studies have found that cardiomyocyte EV increase in the blood stream after experimental myocardial infarction and after exposure of cardiomyocytes to hypoxic conditions (56). *In-vitro* studies revealed that they can exert numerous biological effects, e.g., modulation of endothelial function, regulation of local inflammatory responses and cardiac remodeling and promotion of hepatic C-reactive protein expression (24, 26, 43, 57, 58).

Their intriguing characteristics predispose cardiomyocyte EV as potential prognostic biomarkers after myocardial infarction. Myocardial infarction severity in a clinical setting is often quantified by echocardiography and an LVEF <40% is commonly associated with larger infarct areas and poor prognosis (59–61). We demonstrate, for the first time, that caveolin-3^+^ EV were increased in STEMI patients with predicted poor outcome (LVEF <40%), compared to patients with better outcome (LVEF ≥40%). Additionally, caveolin-3^+^ EV correlated with CK-MB_max_, a known marker of myocardial infarction severity (32). Caveolin-3^+^ EV also correlated with time from reperfusion to blood sampling, indicating a time dependent release, previously reported for conventional biomarkers of myocardial infarction (62). This time-dependent release after myocardial infarction further supports our hypothesis, that caveolin-3^+^ EV were released in response to ischemic myocardial injury and that caveolin-3^+^ EV are increased at least during the first day after myocardial infarction.

Cardiomyocyte EV were recently explored as biomarkers in patients with ischemic cardiomyopathies and aortic stenosis. Anselmo et al. used CD172a as marker for cardiomyocyte EV (63). Interestingly, higher levels of CD172a^+^ cardiomyocyte EV were associated with a favorable prognosis after transcatheter aortic valve replacement. In light of our findings, this could indicate that there are distinct cardiomyocyte EV populations, which are associated with either a favorable (CD172a^+^) or poor (caveolin-3^+^) prognosis. These empirical findings might also be related to the biological effects of cardiomyocyte EV. For example, in Anselmo et al.'s study, CD172a^+^ cardiomyocyte EV acted in a beneficial sense by promoting contraction of isolated cardiomyocytes, whereas caveolin-3^+^ EV have been known to exert harmful effects, e.g., increased secretion of pro-inflammatory cytokines, such as IL-6 and CCL-2 (26).

Levels of leukocyte (CD45^+^) EV did not differ significantly in VA-ECMO and STEMI patients. This is most likely due to the fact that increased levels of leukocyte EV have been reported in both ECMO (10, 38) and STEMI patients (42). Interestingly, the level of leukocyte EV was increased in non-survivors of VA-ECMO therapy indicating an association with outcome. ECMO may result in initial leukocyte activation and EV release, followed by leukocyte dysfunction, previously associated with poor outcome (9, 29, 64–66), which may explain increased levels of leukocyte EV in non-survivors of VA-ECMO therapy. Moreover, leukocyte EV may also be exerting harmful biological effects explaining their association with outcome. For example, monocyte-derived EV are able to induce upregulation of adhesion receptors potentially increasing thrombogenicity (67, 68), which could predispose VA-ECMO patients to thrombotic complications (although they occur less frequently than bleeding complications). Additionally, neutrophil EV have been shown to upregulate of IL-6 in endothelial cells (69), which could further contribute to an initial overshooting inflammatory response in VA-ECMO patients associated with poor outcome (8, 67, 68).

We also found that leukocyte EV correlated with the number coronary stents placed during the acute coronary angiography in STEMI patients. CD45^+^ EV are released from activated leukocytes (70) and laboratory investigations indicate that coronary stents can activate leukocytes (71). Moreover, clinical studies have reported leukocyte activation in patients receiving coronary stents (72). Hence, leukocyte activation triggered by placement of coronary stents may be the mechanism behind the observed correlation between the number of coronary stents placed and increasing levels of leukocyte EV.

## Strengths and Limitations

Since only few studies have investigated EV in ECMO patients, this study expands the existing knowledge in this field. By comparing EV levels from ECMO to *STEMI patients*, we provide more realistic controls than, for example, healthy volunteers. Furthermore, our study indicates that EV may be used as biomarkers to predict outcome in these patients. In STEMI patients, we identify an intriguing EV population, caveolin-3^+^ EV, which were associated with outcome.

However, due to its observational nature we can only report associations between EV populations and disease states. On a methodological level, we analyzed EV with a size from 100 nm to 1 μm. Therefore, we cannot comment on the role of smaller or larger EV. As we only sampled blood from VA-ECMO patients at one timepoint, we cannot determine how the initiation and continuation of VA-ECMO therapy influenced the EV profile. Moreover, VA-ECMO patients had different underlying diseases, which might have influenced results. However, a subgroup analysis of EV from VA-ECMO patients with and without CAD showed similar results in both groups indicating that the influence of VA-ECMO therapy potentially outweighs the influence of the underlying disease.

Since we used STEMI patients as controls, it may have been advantageous if the group of VA-ECMO patients featured more STEMI patients. As this was difficult to achieve in practice, we analyzed EV levels in a more homogeneous VA-ECMO subgroup: those with known CAD. EV levels in this subgroup were compared to the group of STEMI patients. Interestingly, we found that the significant differences regarding EV levels remained the same as previously observed compared to the whole group. These results emphasize the influence of VA-ECMO therapy on EV levels, whereas the influence of the underlying disease may be limited.

This study primarily aimed to investigate the association of caveolin-3^+^ EV with outcome but future studies are required to characterize their biological role in detail.

## Conclusion

The EV profile in VA-ECMO patients differs from STEMI patients in several regards. Levels of total Annexin V^+^ EV are lower in VA-ECMO patients than in STEMI patients, but the percentage of CD61^+^ platelet EV is higher. Increased levels of total Annexin V^+^ EV in VA-ECMO patients on day 1 are predictive of mortality. Total Annexin V^+^ EV may therefore serve as novel prognostic biomarkers guiding early clinical decision making for VA-ECMO patients in the future. Caveolin-3^+^ cardiomyocyte EV, both absolute counts and the percentage of Annexin V^+^ EV, were significantly higher in STEMI patients. Caveolin-3^+^ EV were related to infarct size and associated with poor outcome in STEMI patients as levels were increased in patients with moderately-severely reduced LVEF and correlated with CK-MB_max_. In the future, caveolin-3^+^ EV may aid clinicians in the early identification of STEMI patients at risk of poor outcome. Further studies with larger patient numbers are needed to elucidate their true capacity as prognostic biomarkers.

## Data Availability Statement

The raw data supporting the conclusions of this article will be made available by the authors upon reasonable request.

## Ethics Statement

The studies involving human participants were reviewed and approved by Ethics Committee of the University of Freiburg. The patients/participants provided their written informed consent to participate in this study.

## Author Contributions

PS: study design, acquisition, analysis and interpretation of data, and preparation of manuscript. IB and JC: acquisition and analysis of data. LH and MR: analysis and interpretation of data and preparation of manuscript. GT, TW, and CB: study design and interpretation of data. DD: study design, interpretation of data, and preparation of manuscript. PD: study design, analysis and interpretation of data, and preparation of manuscript. All authors proof-read and accepted the final draft of the manuscript.

## Funding

This work was funded by institutional grants from the University of Freiburg. DD is a member of SFB1425, funded by German Research Foundation (DFG; Project #422681845). LH received funding from the German Research Foundation (DFG; HE 8679/1-1:1). The article processing charge was funded by the Baden-Wuerttemberg Ministry of Science, Research and Art and the University of Freiburg in the funding program Open Access Publishing.

## Conflict of Interest

The authors declare that the research was conducted in the absence of any commercial or financial relationships that could be construed as a potential conflict of interest.

## Publisher's Note

All claims expressed in this article are solely those of the authors and do not necessarily represent those of their affiliated organizations, or those of the publisher, the editors and the reviewers. Any product that may be evaluated in this article, or claim that may be made by its manufacturer, is not guaranteed or endorsed by the publisher.
