# Supplementary material for: Extracellular Vesicles Are Associated With Outcome in Veno-Arterial Extracorporeal Membrane Oxygenation and Myocardial Infarction
Source: Front Cardiovasc Med. 2021 Nov 4;8:747453. doi: 10.3389/fcvm.2021.747453 (PMC8600355; doi:10.3389/fcvm.2021.747453)
Supplement: Supplementary file 1 [file Data_Sheet_1.pdf]

Parameter	STEMI patients (n=19)
No. of diseased vessels	
- 1	7 (37)
- 2	7 (37)
- 3	5 (26)
No. of stents implanted	
- 1	9 (47)
- 2	6 (32)
- 3	3 (16)
- 4	1 (5)
Anti-platelet agents: ASA +	
- Clopidogrel	1 (5)
- Ticagrelor	4 (21)
- Prasugrel	14 (74)
Time from reperfusion to blood sampling (h, Q1-Q3)	12 (10-15)

Supplementary Table S1. Specific clinical characteristics of STEMI patients. Data are presented as median (interquartile range, Q1-Q3) or number of patients (%). Denominator of the percentage is the total number of subjects in the group. ASA=acetylsalicylic acid.

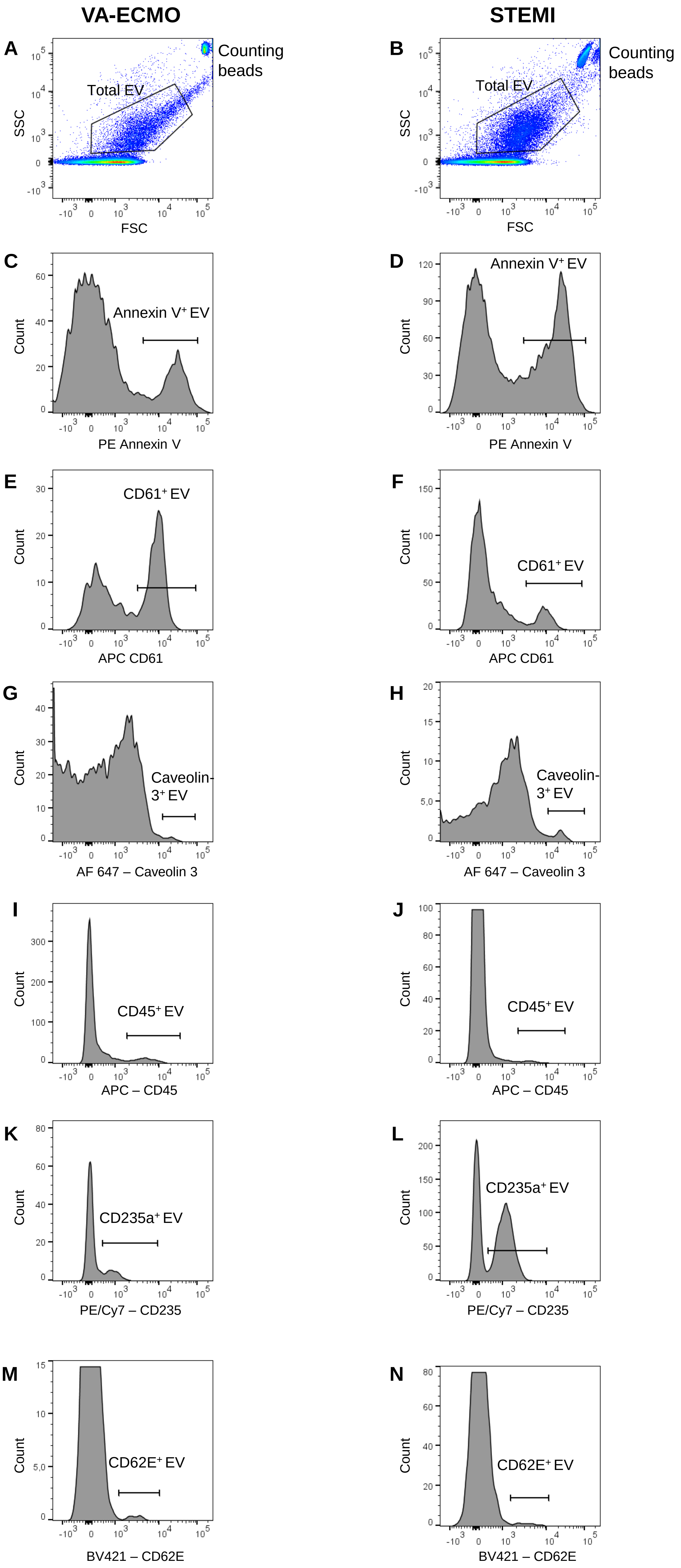

Supplementary Figure S1

Supplementary Figure S1. Representative dot plots and histograms of extracellular vesicle (EV) populations in VA-ECMO and STEMI patients identified by flow cytometry. Sample preparation, staining and data acquisition were performed as described in the Materials and Methods section. **A, B** Total EV were identified by size reference beads similar to Jayachandran et al (1). Counting beads (BD Trucount™) shown in the upper right hand corner of the dot plot were used to calculate absolute EV counts. **C, D** From Total EV, Annexin V^+ EV were identified by PE Annexin V^+. A clear Annexin V^+ population was gated. This Annexin V^+ population is also referred to as 'total Annexin V^+ EV' in the manuscript. **E-N** $CD61^+$ (platelet), caveolin-3^+ (cardiomyocyte), $CD45^+$ (leukocyte), $CD235a^+$ (erythrocyte) and $CD62E^+$ (endothelial) EV were identified with the help of specific antibodies. These populations were subpopulatations of the Annexin V^+ population. Samples stained with corresponding isotype controls were used to set the gate. Populations of $CD45^+$ and $CD62E^+$ were relatively small, particularly in the STEMI group.

References

1. Jayachandran M, Miller VM, Heit JA, Owen WG. Methodology for isolation, identification and characterization of microvesicles in peripheral blood. J Immunol Methods (2012) 375:207-14.https://doi.org/10.1016/j.jim.2011.10.012

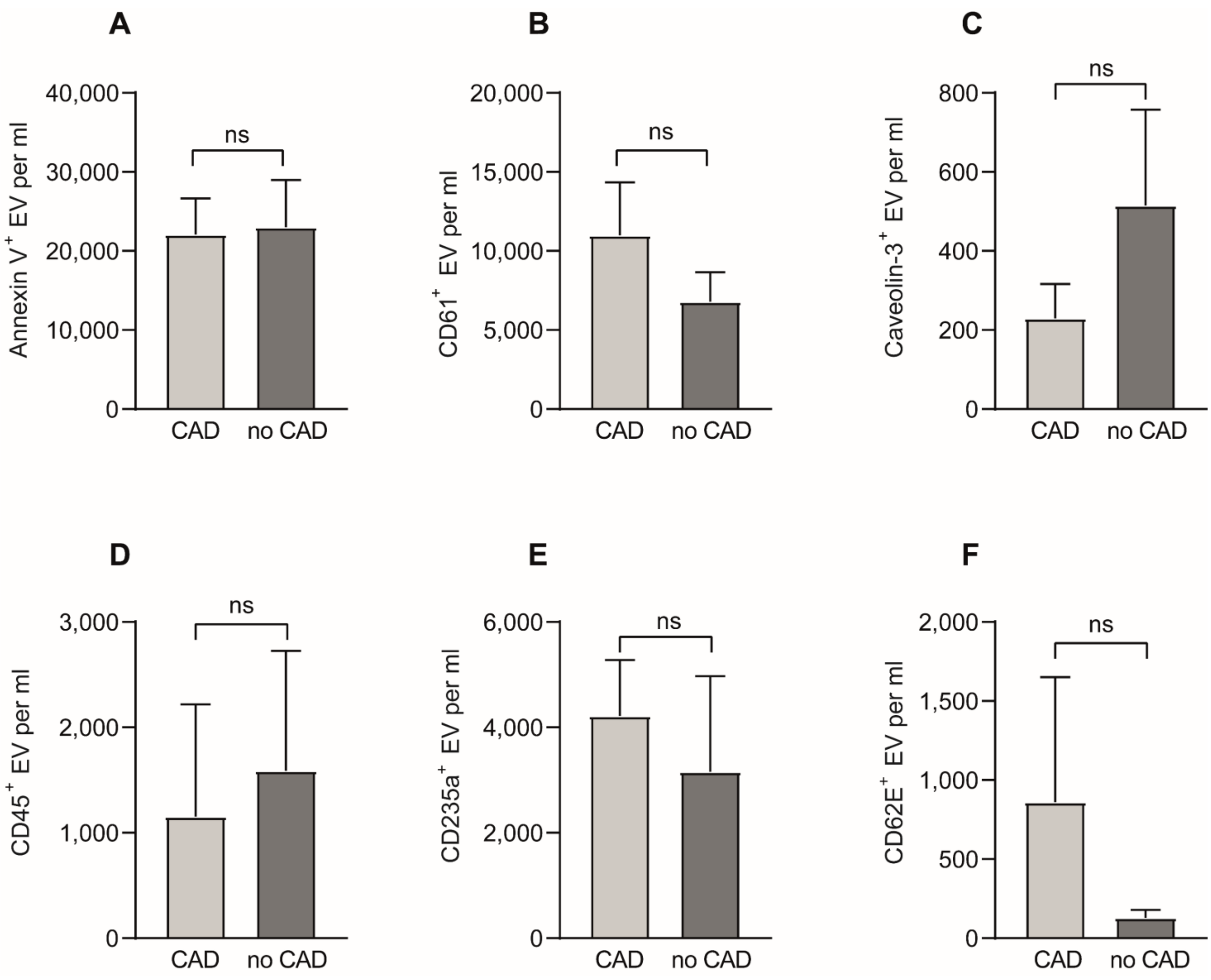

Supplementary Figure S2. Extracellular vesicle (EV) concentration in patients receiving veno-arterial extracorporeal membrane oxygenation with coronary artery disease (CAD) vs. without CAD (no CAD). (**A**) Total Annexin V^+ EV/ml, (**B**) $CD61^+$ (platelet) EV/ml, (**C**) caveolin-3^+ (cardiomyocyte) EV/ml, (**D**) $CD45^+$ (leukocyte) EV/ml, (**E**) $CD235a^+$ (erythrocyte) EV/ml, (**F**) $CD62E^+$ (endothelial) EV/ml. EV isolation, analysis and quantification were performed as described in the Materials and Methods section. Data are presented as mean±SEM. CAD: n=11; no CAD: n=7. p-values were calculated by an unpaired t-test, $p \leq 0.05$ was considered significant, ns – not significant.

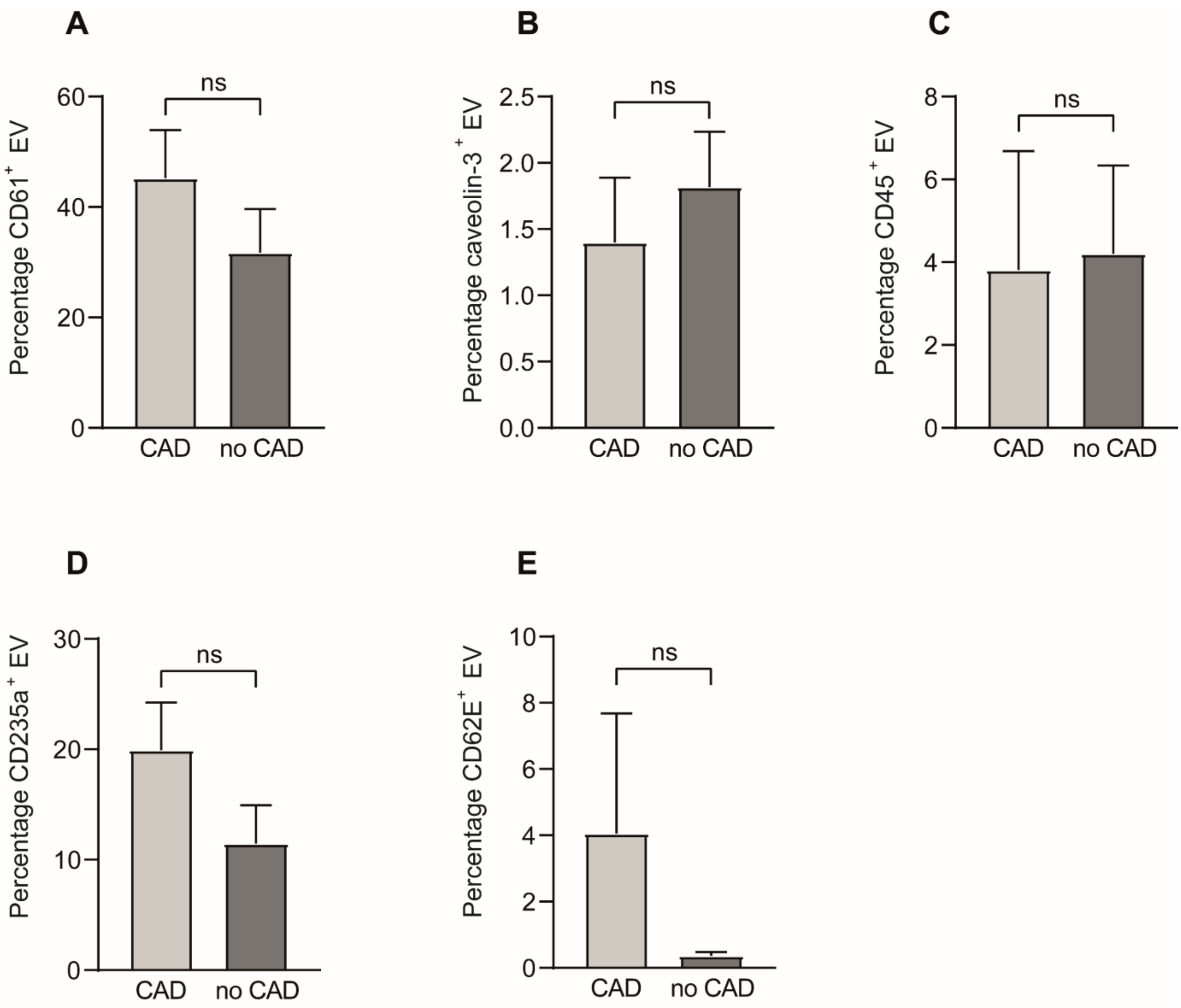

Supplementary Figure S3. Extracellular vesicles (EV) as percentage of Annexin V^+ EV from patients receiving veno-arterial extracorporeal membrane oxygenation with coronary artery disease (CAD) vs. without CAD (no CAD). (**A**) $CD61^+$ (platelet) EV, (**B**) caveolin-3^+ (cardiomyocyte) EV, (**C**) $CD45^+$ (leukocyte) EV, (**D**) $CD235a^+$ (erythrocyte) EV, (**E**) $CD62E^+$ (endothelial) EV. EV isolation, analysis and quantification were performed as described in the Materials and Methods section. Data are presented as mean±SEM. CAD: n=11; no CAD: n=7. p-values were calculated by an unpaired t-test, $p \leq 0.05$ was considered significant, ns – not significant.

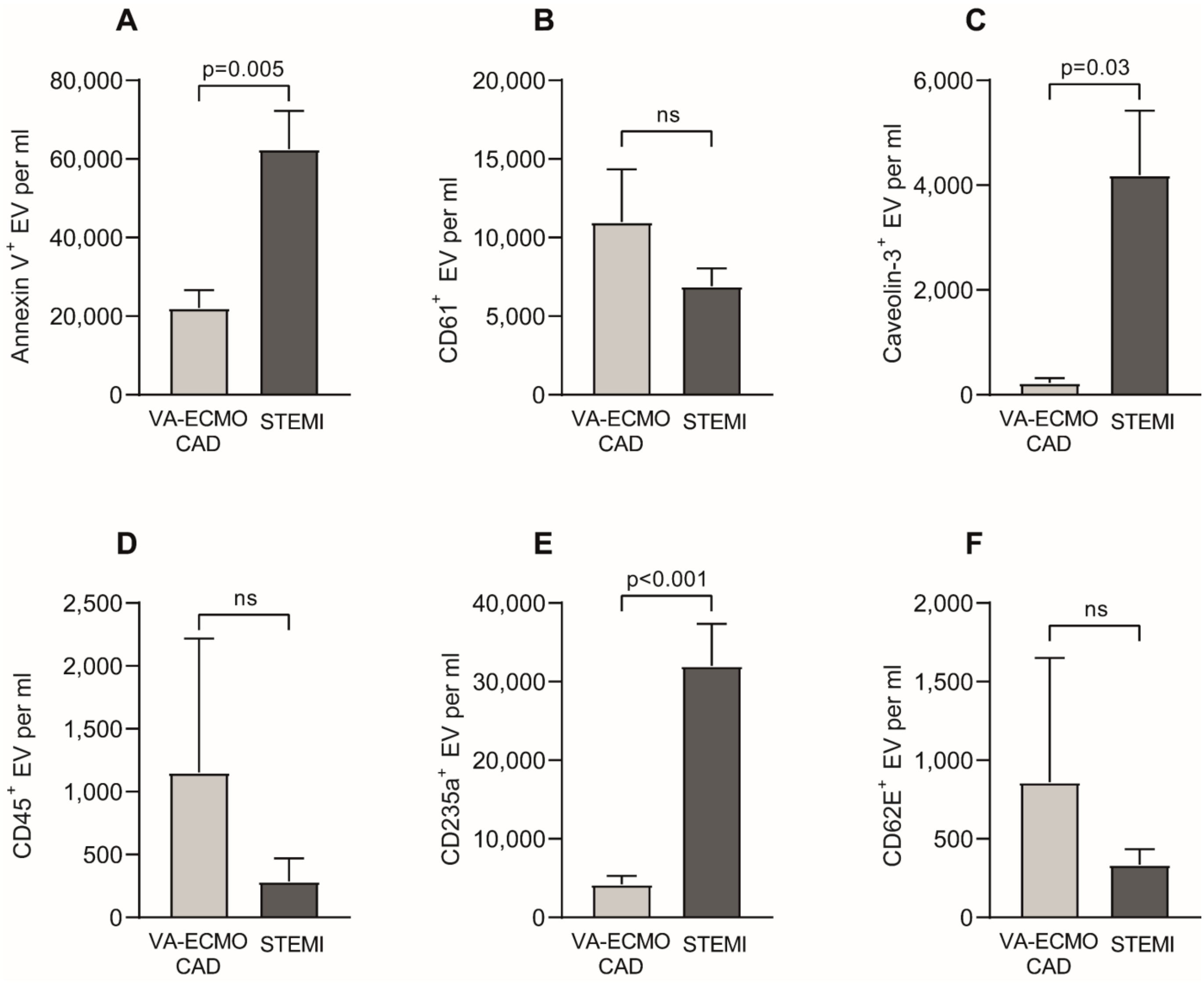

Supplementary Figure S4. Extracellular vesicle (EV) concentration in patients receiving veno-arterial extracorporeal membrane oxygenation with coronary artery disease (VA-ECMO CAD) vs. STEMI patients (STEMI). (**A**) Total Annexin V^+ EV/ml, (**B**) $CD61^+$ (platelet) EV/ml, (**C**) caveolin-3^+ (cardiomyocyte) EV/ml, (**D**) $CD45^+$ (leukocyte) EV/ml, (**E**) $CD235a^+$ (erythrocyte) EV/ml, (**F**) $CD62E^+$ (endothelial) EV/ml. EV isolation, analysis and quantification were performed as described in the Materials and Methods section. Data are presented as mean±SEM. VA-ECMO CAD: n=11; STEMI: n=19. p-values were calculated by an unpaired t-test, $p \leq 0.05$ was considered significant, ns – not significant.

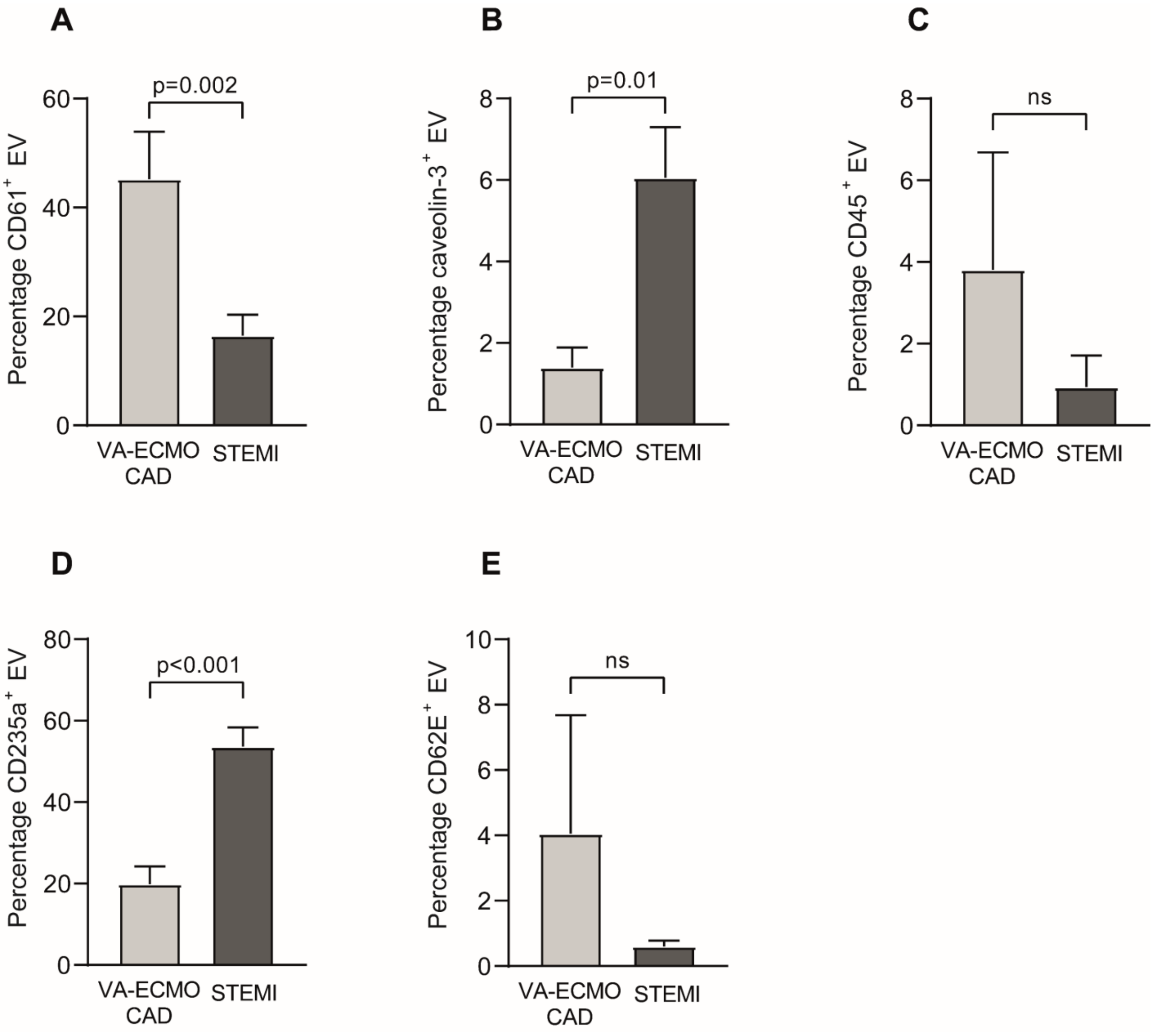

Supplementary Figure S5. Extracellular vesicles (EV) as percentage of Annexin V^{+} EV from patients receiving veno-arterial extracorporeal membrane oxygenation with coronary artery disease (VA-ECMO CAD) vs. STEMI patients (STEMI). (**A**) $CD61^{+}$ (platelet) EV, (**B**) caveolin-3^{+} (cardiomyocyte) EV, (**C**) $CD45^{+}$ (leukocyte) EV, (**D**) $CD235a^{+}$ (erythrocyte) EV, (**E**) $CD62E^{+}$ (endothelial) EV. EV isolation, analysis and quantification were performed as described in the Materials and Methods section. Data are presented as mean±SEM. VA-ECMO CAD: n=11; STEMI: n=19. p-values were calculated by an unpaired t-test, p≤0.05 was considered significant, ns – not significant.

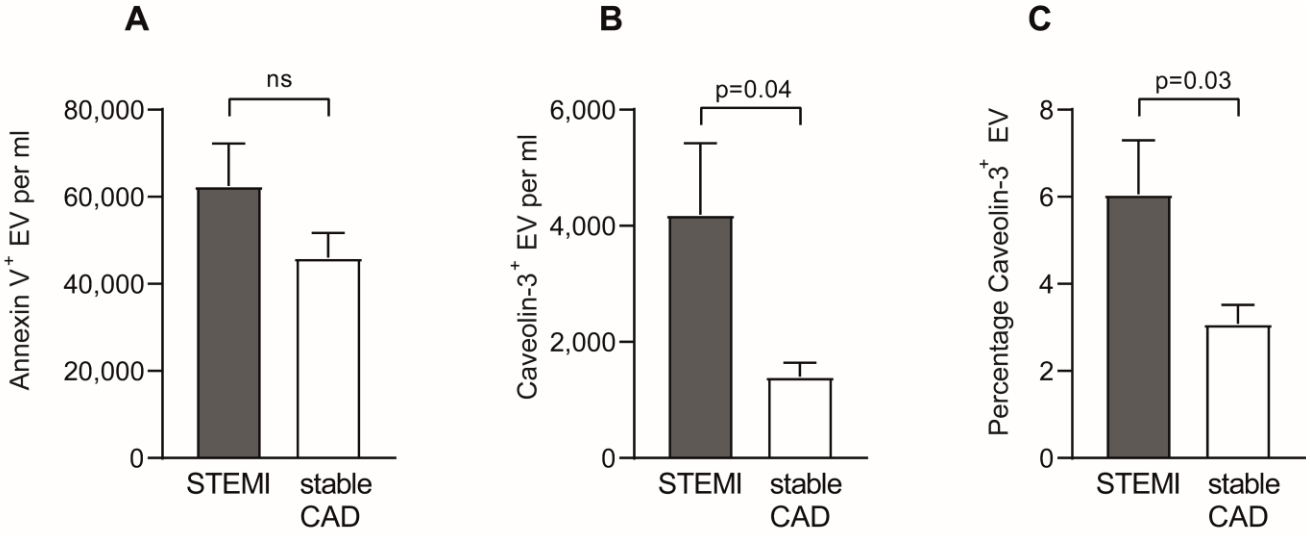

Supplementary Figure S6. Extracellular vesicle (EV) concentration and percentage of Annexin V$^+$ EV in STEMI patients vs. patients with stable coronary artery disease (CAD). (**A**) Annexin V$^+$ EV/ml, (**B**) Caveolin-3$^+$ (cardiomyocyte) EV/ml, (**C**) Caveolin 3$^+$ EV in percentage of Annexin V$^+$ EV. 10 patients with stable coronary artery disease were recruited 2-7 h after routine coronary angiography. Coronary angiography confirmed stable coronary artery disease and no stents were placed. Exclusion criteria were: Age<18 or >80 years, hematological malignancies, hemoglobin (Hb)-values under 8 g/dl, Sepsis (as defined by positive blood cultures). Median patient age was 67 years (52-77). Of the 10 patients with CAD, 4 patients were females, 6 were males. 4 patients were smokers, 8 patients had arterial hypertension, 9 patients suffered from hypercholesterolemia, 3 patients had a family history of CAD and 5 patients had Diabetes mellitus. Annexin V$^+$ EV levels did not significantly differ between groups but caveolin-3$^+$ EV per ml and in percentage were significantly higher in the STEMI group. EV isolation, analysis and quantification of caveolin-3$^+$ EV were performed as described in the Materials and Methods section. Data are presented as mean±SEM. STEMI patients: n=19, stable CAD patients: n=10. p-values were calculated by an unpaired t-test, $p \leq 0.05$ was considered significant, ns – not significant.
